# Detecting a Photon-Number Splitting Attack in Decoy-State Measurement-Device-Independent Quantum Key Distribution via Statistical Hypothesis Testing

**DOI:** 10.3390/e24091232

**Published:** 2022-09-02

**Authors:** Xiaoming Chen, Lei Chen, Yalong Yan

**Affiliations:** 1 School of Cyberspace Security, Beijing University of Posts and Telecommunications, Beijing 100876, China; 2 Beijing Electronic Science and Technology Institute, Beijing 100070, China; 3 School of Cyber Science and Technology, University of Science and Technology of China, Hefei 230026, China

**Keywords:** decoy state, measurement-device independent, quantum key distribution, photon number splitting attack, statistical hypothesis testing

## Abstract

Measurement-device-independent quantum key distribution (MDI-QKD) is innately immune to all detection-side attacks. Due to the limitations of technology, most MDI-QKD protocols use weak coherent photon sources (WCPs), which may suffer from a photon-number splitting (PNS) attack from eavesdroppers. Therefore, the existing MDI-QKD protocols also need the decoy-state method, which can resist PNS attacks very well. However, the existing decoy-state methods do not attend to the existence of PNS attacks, and the secure keys are only generated by single-photon components. In fact, multiphoton pulses can also form secure keys if we can confirm that there is no PNS attack. For simplicity, we only analyze the weaker version of a PNS attack in which a legitimate user’s pulse count rate changes significantly after the attack. In this paper, under the null hypothesis of no PNS attack, we first determine whether there is an attack or not by retrieving the missing information of the existing decoy-state MDI-QKD protocols via statistical hypothesis testing, extract a normal distribution statistic, and provide a detection method and the corresponding Type I error probability. If the result is judged to be an attack, we use the existing decoy-state method to estimate the secure key rate. Otherwise, all pulses with the same basis leading to successful Bell state measurement (BSM) events including both single-photon pulses and multiphoton pulses can be used to generate secure keys, and we give the formula of the secure key rate in this case. Finally, based on actual experimental data from other literature, the associated experimental results (e.g., the significance level is 5%) show the correctness of our method.

## 1. Introduction

Quantum key distribution (QKD) [1,2,3,4,5,6] is a technique that allows two remote parties (Alice and Bob), to share unconditional secure keys. The unconditional security of the keys are guaranteed by the laws of quantum mechanics [7,8,9,10]. The first ideal QKD protocol is BB84-QKD created by Bennett and Brassard [1], which needs a perfect single-photon source and detectors. However, there is always a large gap between ideal and reality. Due to the imperfection of equipment, the implementation of the QKD suffers double attacks from the source side and detection side. On the one hand, at present, perfect single-photon sources are not available, and weak coherent photon sources (WCPs) after phase randomization are often utilized to replace the single-photon sources. While the photon number of the pulses emitted by WCPs may be more than one, an eavesdropper Eve can launch a photon-number splitting (PNS) attack [11,12,13,14,15]. Specially, a weaker version of a PNS attack is one in which Alice’s or Bob’s pulse count rate changes significantly after the attack [11,12,13,14], and the stronger PNS attack means that both Alice’s and Bob’s pulse count rates remain unchanged after the attack [15]. The difference between these two attacks is the effect on Alice’s and Bob’s pulse count rates. Fortunately, the decoy-state method [16,17,18] proposed later can resist PNS attacks very well.

On the other hand, due to the low detection efficiency of the detectors, Eve can launch attacks against the detectors. Compared with source attacks, there are more attacks from the detection side, such as the detector blinding attack [19,20], dead time attack [21], faked state attack [22,23], and time shift attack [24].People have proposed device-independent quantum key distribution (DI-QKD) [25,26], which can resist all attacks from devices. However, this protocol is highly impractical because it needs close to unity detection efficiency. In 2012, Lo et al. [27] proposed measurement-device-independent quantum key distribution (MDI-QKD), which is also known as the time-inversion version of EPR protocol [28]. In MDI-QKD, Alice and Bob do not need to perform measurement operations, so it can be innately immune to all detection attacks. MDI-QKD combined with the decoy-state method can resist both source attacks and detection attacks; thus, decoy-state MDI-QKD [29,30,31] is one of the most promising QKD protocols, which can provide unconditional secure keys in practical applications.

However, the secure key rate of the existing decoy-state MDI-QKD is not high [32,33]. The decoy-state method defeats the PNS attack through providing a more accurate method to determine the secure key rate. More specifically, the existing decoy-state method can more closely estimate the lower bound of gain and the upper bound of quantum bit error rate (QBER) of single-photon signals, and then the secure key rate can be calculated by the GLLP formula [34]. In essence, the existing decoy-state method does not care about the existence of a PNS attack, and the secure keys are only generated by single-photon components [35]. However, if we can determine that there is no PNS attack on the channel, multiphoton pulses can also generate secure keys. For simplicity, we only analyze the weaker version of PNS attack in which the legitimate user’s pulse count rate changes significantly after the attack. In this case, there is no doubt that using the existing methods to estimate the secure key rate will waste the underlying keys generated from multiphoton pulses and reduce the efficiency.

In this work, under the null hypothesis of no PNS attack $H_{0}$, we first retrieve the lost information in the existing decoy-state MDI-QKD, extract a normal distribution statistic, and provide a new method to determine whether there is a PNS attack or not through statistical hypothesis testing. If the result is judged to be an attack, the keys can only be generated from single-photon pulses, and the secure key rate will be estimated by the existing decoy-state method. Otherwise, all pulses with the same basis leading to a successful Bell state measurement (BSM) event including both single-photon pulses and multiphoton pulses can be used to generate keys, and we give the formula of the secure key rate in this case. Furthermore, we use the real experimental data in [36] to verify our method, and the analytical results show that our method is credible (e.g., a significance level of 5%).

The structure of this paper is organized as follows. In Section 2, we briefly review the typical decoy-state MDI-QKD and related notations. In Section 3, we describe our method for detecting the PNS attack in the decoy-state MDI-QKD via statistical hypothesis testing in detail. In Section 4, the correctness of our method is verified with the real experimental data from the existing literature. Finally, we discuss and draw conclusions in Section 5.

## 2. Three-Intensity Decoy-State MDI-QKD

In this paper, we adopt a typical decoy-state MDI-QKD with polarization encoding [36], which mainly consists of three steps.

(i) Alice generates phase-randomized pulses from WCPs and randomly selects the basis W∈{Z,X}. That is, $P_{Z}=P_{X}=1/2$, where $P_{Z}$ and $P_{X}$ are the probabilities of choosing the *Z* basis and *X* basis, respectively. Then Alice uses an intensity modulator to modulate the pulses with three different intensities and sends them to Charlie located in the middle. This three intensities are the intensity of signal state $\mu_{2}$, the intensity of decoy state $\mu_{1}$, and the intensity of vacuum state $\mu_{0}$, respectively. Furthermore, the corresponding percentages being emitted are $P_{\mu_{2}}$, $P_{\mu_{1}}$, and $P_{\mu_{0}}$, respectively. Obviously, $P_{\mu_{2}}+P_{\mu_{1}}+P_{\mu_{0}}=1$. At the same time, Bob performs the same procedures as Alice, and the intensities of Bob’s pulses are noted as $\nu_{2}$, $\nu_{1}$, and $\nu_{0}$ for the signal state, decoy state, and vacuum state, respectively. Similarly, the corresponding percentages being emitted are $P_{\nu_{2}}$, $P_{\nu_{1}}$, and $P_{\nu_{0}}$, respectively, where $P_{\nu_{2}}+P_{\nu_{1}}+P_{\nu_{0}}=1$.

(ii) The pulses from Alice and Bob interfere when they reach Charlie. Then Charlie performs a Bell state measurement (BSM) on the interference outcomes and announces the measurement results to Alice and Bob.

(iii) Alice and Bob compare their bases, and determine the secure keys through Charlie’s measurement results. Specifically, if Alice and Bob choose the same basis and Charlie has a successful BSM event at the same time, then this part of the pulses can generate keys. It is important to emphasize that the secure keys are only generated from the signal state with *Z* basis, and the others are used for parameter estimation.

The secure key rate of the decoy-state MDI-QKD [27,36] is given by

(1) $$R\geq q\{P_{11}^{\mu_{2}\nu_{2}}Y_{11}^{Z}\left[1-H\left(e_{11}^{X}\right)\right]-Q_{\mu_{2}\nu_{2}}^{Z}f_{e}H\left(E_{\mu_{2}\nu_{2}}^{Z}\right)\}.$$

In the above equation, $q=P_{Z}^{2}P_{\mu_{2}}P_{\nu_{2}}$ is the probability that Alice and Bob both select the *Z* basis and both modulate the pulse as signal state. $P_{11}^{\mu_{2}\nu_{2}}=\mu_{2}\nu_{2}e^{-\mu_{2}-\nu_{2}}$ is the probability that the pulses from Alice’s signal state and Bob’s signal state are both single-photon pulses. $Y_{11}^{Z}$ and $e_{11}^{X}$ are the yield of single-photon state with *Z* basis and the quantum bit error rate (QBER) of single-photon state with *X* basis. $H\left(x\right)=-x\log_{2}\left(x\right)-\left(1-x\right)\log_{2}\left(1-x\right)$ is the binary Shannon entropy function. $Q_{\mu_{2}\nu_{2}}^{Z}$ and $E_{\mu_{2}\nu_{2}}^{Z}$ are the overall gain and overall QBER of signal state with *Z* basis, respectively. $f_{e}>1$ is the error correction efficiency.

According to [37,38], the overall gain $Q_{\mu_{k}\nu_{l}}^{W}$ (W∈{X,Z}) and the overall QBER $E_{\mu_{k}\nu_{l}}^{W}$ (W∈{X,Z}) can be obtained by the following equations,

(2) $$\begin{array}{cc} Q_{\mu_{k}\nu_{l}}^{X} & =2y^{2}\left[1+2y^{2}-4yI_{0}\left(x\right)+I_{0}\left(2x\right)\right],\\ E_{\mu_{k}\nu_{l}}^{X}Q_{\mu_{k}\nu_{l}}^{X} & =e_{0}Q_{\mu_{k}\nu_{l}}^{X}-2\left(e_{0}-e_{d}\right)y^{2}\left[I_{0}\left(2x\right)-1\right],\\ Q_{\mu_{k}\nu_{l}}^{Z} & =Q_{C}+Q_{E},\\ E_{\mu_{k}\nu_{l}}^{Z}Q_{\mu_{k}\nu_{l}}^{Z} & =e_{d}Q_{C}+\left(1-e_{d}\right)Q_{E}. \end{array}$$

where

(3) $$\begin{array}{cc} Q_{C} & =2{\left(1-p_{d}\right)}^{2}e^{-\mu^{\prime }/2}\left[1-\left(1-p_{d}\right)e^{-\eta_{a}\mu_{k}/2}\right]\times \left[1-\left(1-p_{d}\right)e^{-\eta_{b}\nu_{l}/2}\right],\\ Q_{E} & =2p_{d}{\left(1-p_{d}\right)}^{2}e^{-\mu^{\prime }/2}\left[I_{0}\left(2x\right)-\left(1-p_{d}\right)e^{-\mu^{\prime }/2}\right]. \end{array}$$

In the above equations, $\mu_{k}$ and $\nu_{l}$, k,l∈{0,1,2}, are the intensities of pulses emitted by Alice and Bob, respectively. $I_{0}\left(x\right)$ is the modified Bessel function of the first kind. $e_{0}$ is the error rate of background. $e_{d}$ is the misalignment-error probability. $p_{d}$ is the dark count rate. $\eta_{a}$ and $\eta_{b}$ are the transmission efficiencies of Alice and Bob, respectively. In addition,

(4) $$\begin{array}{cc} x & =\sqrt{\eta_{a}\mu_{k}\eta_{b}\nu_{l}}/2,\\ y & =\left(1-p_{d}\right)e^{-\mu^{\prime }/4},\\ \mu^{\prime } & =\eta_{a}\mu_{k}+\eta_{b}\nu_{l},\\ \eta_{a} & =\eta_{d}10^{-\frac{\delta L_{ac}+\theta }{10}},\\ \eta_{b} & =\eta_{d}10^{-\frac{\delta L_{bc}+\theta }{10}}, \end{array}$$

where $\eta_{d}$ is the quantum efficiency of detectors, δ is the loss coefficient measured in dB/km, $L_{ac}$ $\left(L_{bc}\right)$ is the distance in km from Alice (Bob) to Charlie, and θ is the insertion loss in Charlie’s measurement setup in dB. Without Eve’s intervention, based on Equations (2)–(4), the yield and the QBER of single-photon pulses when Alice and Bob select the same basis *X* or *Z* are, respectively, given by

(5) $$\begin{array}{cc} Y_{11}^{X} & =Y_{11}^{Z}={\left(1-p_{d}\right)}^{2}\left[\frac{\eta_{a}\eta_{b}}{2}+\left(2\eta_{a}+2\eta_{b}-3\eta_{a}\eta_{b}\right)p_{d}+4\left(1-\eta_{a}\right)\left(1-\eta_{b}\right)p_{d}^{2}\right],\\ e_{11}^{X}Y_{11}^{X} & =e_{0}Y_{11}^{X}-\left(e_{0}-e_{d}\right){\left(1-p_{d}\right)}^{2}\frac{\eta_{a}\eta_{b}}{2},\\ e_{11}^{Z}Y_{11}^{Z} & =e_{0}Y_{11}^{Z}-\left(e_{0}-e_{d}\right){\left(1-p_{d}\right)}^{2}\left(1-p_{d}\right)\frac{\eta_{a}\eta_{b}}{2}. \end{array}$$

## 3. Statistical Hypothesis Testing

In this section, we introduce a new method to detect the PNS attack in the decoy-state MDI-QKD via statistical hypothesis testing. It is important to emphasize that the PNS attacks mentioned here and below refer to the weaker version of PNS attack. Then we analyze the Type I error of the test; that is, mistaking no PNS attack when there is a PNS attack. Generally speaking, our method first puts forward a null hypothesis and alternative hypothesis based on the theory of statistical hypothesis testing. Then, the test statistic is constructed according to the null hypothesis and other conditions. Furthermore, the specific values of the statistics can be obtained by using the parameters and experimental data. After the significance level is given, we can infer whether there is PNS attack in the channel with a certain probability. The details are as follows.

(i) Identify null and alternative hypothesis. Let us consider the hypothesis testing problem of the null hypothesis $H_{0}$: there is no PNS attack on the channel and the alternative hypothesis $H_{1}$: there is a PNS attack on the channel.

(ii) Construct the test statistic. We need a test statistic to conduct the hypothesis testing. In what follows, the distribution of the test statistic is derived under the null hypothesis $H_{0}$. Let us further consider Alice’s and Bob’s pulses emission process and Charlie’s BSM event. When Alice and Bob send pulses with the same basis, the BSM event outcomes at Charlie only include two cases, successful or failed. Therefore, the above process can be regarded as a Bernoulli trial. Note that $Q_{\mu_{k}\nu_{l}}^{W}$ is the probability that Charlie obtains a successful BSM event provided that Alice and Bob emit pulses with the intensities $\mu_{k}$ and $\nu_{l}$ and select the basis *W*. Suppose the total number of pulses emitted by Alice (Bob) is $N_{data}$, then the number of pulses is $P_{W}^{2}P_{\mu_{k}\nu_{l}}N_{data}$ when Alice’s and Bob’s intensities with *W* basis are $\mu_{k}$ and $\nu_{l}$, respectively. In the above equation, $P_{W}$ is the probability that Alice (Bob) chooses the W∈{X,Z} basis, $P_{\mu_{k}\nu_{l}}=P_{\mu_{k}}P_{\nu_{l}}$ is the probability that Alice and Bob choose the intensities $\mu_{k}$ and $\nu_{l}$, respectively. At this point, the number of successful BSM events that Charlie obtained is denoted as $n_{\mu_{k}\nu_{l}}^{W}$. Then, $n_{\mu_{k}\nu_{l}}^{W}$ has the binomial distribution with parameters $\left(P_{W}^{2}P_{\mu_{k}\nu_{l}}N_{data},\;Q_{\mu_{k}\nu_{l}}^{W}\right)$, for short,

(6) $$n_{\mu_{k}\nu_{l}}^{W}∼B\left(P_{W}^{2}P_{\mu_{k}\nu_{l}}N_{data},\;Q_{\mu_{k}\nu_{l}}^{W}\right).$$

According to [36], we find $N_{data}$ is so large (typically $10^{10}∼10^{11}$), $Q_{\mu_{k}\nu_{l}}^{W}$ is close to $10^{-8}∼10^{-5}$. Generally, the selections of basis and intensity are random. In other words, $P_{Z}=P_{X}=1/2$, $P_{\mu_{k}}=P_{\nu_{l}}=1/3$ where k,l∈{0,1,2}. Thus, we have $P_{W}^{2}P_{\mu_{k}\nu_{l}}N_{data}Q_{\mu_{k}\nu_{l}}^{W}>P_{W}^{2}P_{\mu_{k}\nu_{l}}N_{data}\left(1-Q_{\mu_{k}\nu_{l}}^{W}\right)\geq 5$. By the law of large numbers and the central limit theorem, when $P_{W}^{2}P_{\mu_{k}\nu_{l}}N_{data}Q_{\mu_{k}\nu_{l}}^{W}\geq 5$ and $P_{W}^{2}P_{\mu_{k}\nu_{l}}N_{data}\left(1-Q_{\mu_{k}\nu_{l}}^{W}\right)\geq 5$, the binomial distribution with parameters $\left(P_{W}^{2}P_{\mu_{k}\nu_{l}}N_{data},\;Q_{\mu_{k}\nu_{l}}^{W}\right)$ can be approximately regarded as the normal distribution with mean $P_{W}^{2}P_{\mu_{k}\nu_{l}}N_{data}Q_{\mu_{k}\nu_{l}}^{W}$ and variance $P_{W}^{2}P_{\mu_{k}\nu_{l}}N_{data}\left(1-Q_{\mu_{k}\nu_{l}}^{W}\right)$, given by

(7) $$n_{\mu_{k}\nu_{l}}^{W}∼N\left(P_{W}^{2}P_{\mu_{k}\nu_{l}}N_{data}Q_{\mu_{k}\nu_{l}}^{W},\;P_{W}^{2}P_{\mu_{k}\nu_{l}}N_{data}\left(1-Q_{\mu_{k}\nu_{l}}^{W}\right)\right).$$

After standardization, we obtain a random variable $U_{\mu_{k}\nu_{l}}^{W}$, which obeys the standard normal distribution; that is,

(8) $$U_{\mu_{k}\nu_{l}}^{W}=\frac{n_{\mu_{k}\nu_{l}}^{W}-P_{W}^{2}P_{\mu_{k}\nu_{l}}N_{data}Q_{\mu_{k}\nu_{l}}^{W}}{\sqrt{P_{W}^{2}P_{\mu_{k}\nu_{l}}N_{data}\left(1-Q_{\mu_{k}\nu_{l}}^{W}\right)}}∼N\left(0,1\right).$$

Considering the additivity of normal distribution, we obtain a random variable involving all possibilities of $U_{\mu_{k}\nu_{l}}^{W}$ where W∈{X,Z}, k,l∈{0,1,2}, which also obeys the normal distribution. There are eighteen cases of $U_{\mu_{k}\nu_{l}}^{W}$ considering that the pair of intensity is nine cases and the selection of basis is two cases. Note that we only consider the same basis for Alice and Bob, that is, both *Z* basis or both *X* basis. After standardization, we obtain a new random variable *V* that obeys the standard normal distribution, which can be written as

(9) $$V=\frac{1}{\sqrt{18}}\sum_{W\in \{Z,X\},\;k,\;l\in \{0,1,2\}}\frac{n_{\mu_{k}\nu_{l}}^{W}-P_{W}^{2}P_{\mu_{k}\nu_{l}}N_{data}Q_{\mu_{k}\nu_{l}}^{W}}{\sqrt{P_{W}^{2}P_{\mu_{k}\nu_{l}}N_{data}\left(1-Q_{\mu_{k}\nu_{l}}^{W}\right)}}∼N\left(0,1\right).$$

Furthermore, Φ(v) is the distribution function of *V*, given by

(10) $$\Phi \left(v\right)=\frac{1}{\sqrt{2\pi }}\int_{-\infty }^{v}e^{-\frac{t^{2}}{2}}dt,\;-\infty <vs.<\infty ,$$

where *v* is the value of *V* and is just the test statistic that we find.

(iii) Find the value of the test statistic. We set the parameters $N_{data}$, $e_{d}$, $e_{0}$, $p_{d}$, $L_{ac}$, $L_{bc}$, δ, θ, $P_{Z}$, $P_{X}$, $\mu_{k}$, $\nu_{l}$, $P_{\mu_{k}}$, and $P_{\nu_{l}}$, where k,l∈{0,1,2}, and we calculate the theoretical value of $Q_{\mu_{k}\nu_{l}}^{W}$ according to Equations (2)–(4). We record $n_{\mu_{k}\nu_{l}}^{W}$ where k,l∈{0,1,2}, W∈{X,Z}. We substitute the above data into Equation (9) and obtain the value of the test statistic *v*.

(iv) Choose a significance level. We need to determine a significance level α (typically 0.05) for the test. In terms of the null hypothesis $H_{0}$ of the test, we deduce that the test is a two-tailed hypothesis testing. Given α, the rejection region is $\left|vs.\right|>v_{\left[1-\alpha /2\right]}$ where $v_{\left[1-\alpha /2\right]}$ can be obtained by Equation (10). More precisely, the variables $-v_{\left[1-\alpha /2\right]}$ and $v_{\left[1-\alpha /2\right]}$ refer to the boundary values between the rejection region and the acceptance region for the test. Let the left side of Equation (10) be equal to α/2; the upper limit of the integral will be $-v_{\left[1-\alpha /2\right]}$. According to the symmetry of the probability density function of normal distribution, $v_{\left[1-\alpha /2\right]}$ can be obtained.

(v) Make a decision. Compare the test statistic *v* with the critical values $v_{\left[1-\alpha /2\right]}$ and $-v_{\left[1-\alpha /2\right]}$. If $v>v_{\left[1-\alpha /2\right]}$ or $v<-v_{\left[1-\alpha /2\right]}$, we will reject $H_{0}$ and accept $H_{1}$. This means that we believe there is a PNS attack on the channel. Otherwise, we fail to reject $H_{0}$. That is to say, we consider there is no PNS attack on the channel. Note that the significance level of the test α is just the Type I error probability of the test, namely, the probability of mistaking no PNS attack for having a PNS attack. Let β denote the Type II error probability of the test, to be precise, the probability of mistaking having a PNS attack for no PNS attack. Note that β is usually difficult to solve in most situations. Furthermore, determining the value of β requires more information about the aggression behavior.

If the result is judged to be a PNS attack, the secure key rate in this case can be estimated by Equation (1). Otherwise, all pulses with the *Z* basis leading to a successful BSM event including both single-photon pulses and multiphoton pulses can be used to generate the keys. Furthermore, the secure key rate formula Equation (1) becomes

(11) $$R\geq qQ_{\mu_{2}\nu_{2}}^{Z}\left[1-f_{e}H\left(E_{\mu_{2}\nu_{2}}^{Z}\right)-H\left(E_{\mu_{2}\nu_{2}}^{Z}\right)\right].$$

By comparing Equation (11) with Equation (1), we can easily find the secure key rate has been highly improved when the judgment result is no PNS attack. This is mainly due to the contribution of multiphoton components.

## 4. Results and Analysis

In the preceding section, we showed the details of our detection method. Now, we move forward to the corresponding experiments based on the aforementioned method and analyze the experimental results. Generally speaking, the real experimental data were substituted into the formulas in Section 3 to verify the correctness of our method. The experimental parameters were from real experiments [36]. Specially, the experimenters in [36] adopted a symmetric scheme; that is, all parameters of Alice and Bob were identical and optimized. The relevant experimental parameters used in [36] and this paper are shown in Table 1.

Based on the above parameters, we can obtain the values of $Q_{\mu_{k}\nu_{l}}^{W}$, as shown in Table 2. Note that Table 2 in this paper is exactly the same as Table I in the Supplementary Materials of [36]. We record the values of $n_{\mu_{k}\nu_{l}}^{W}$, as shown in Table 3. Note that the data in Table 3 can be deduced from Table I in the Main Text of [36]. According to the above data and Equation (8), all values of $U_{\mu_{k}\nu_{l}}^{W}$ can be obtained, as shown in Table 4.

**Figure 1:**
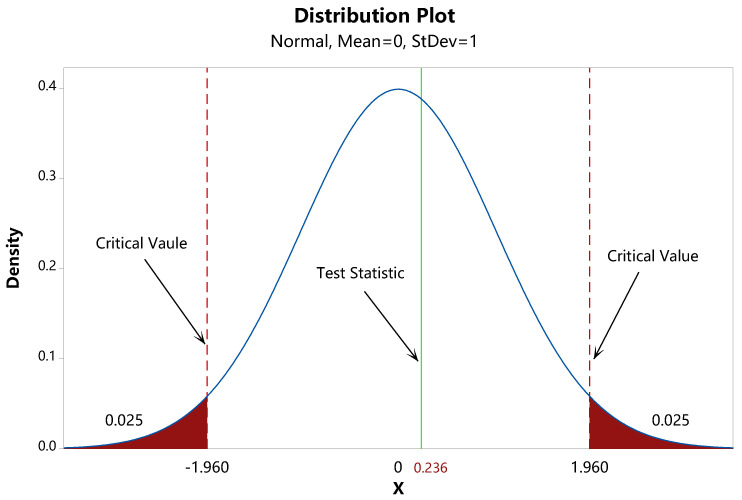
The schematic diagram of statistical hypothesis testing. The value of the test statistic *v* is 0.236. Given the significance level of the test α=0.05, the critical values are $v_{\left[1-\alpha /2\right]}=1.96$ and $-v_{\left[1-\alpha /2\right]}=-1.96$.

The schematic diagram of statistical hypothesis testing is illustrated in Figure 1. After calculation, we obtained the value of the test statistic v=0.236. Given the significance level of the test α=0.05, the critical values were $v_{\left[1-\alpha /2\right]}=1.96$ and $-v_{\left[1-\alpha /2\right]}=-1.96$. Since −1.96<0.236<1.96, the test statistic did not fall inside the rejection region, and we failed to reject $H_{0}$. In other words, we inferred that there was no PNS attack on the channel, and the corresponding Type I error probability was less than 5%. According to [36], there was indeed no PNS attack in the experiment, which verifies the correctness of our method. Thus, both single-photon and multiphoton components can be used to generate keys in this case. At this time, the secure key rate can be estimated through Equation (11).

## 5. Conclusions and Discussion

In summary, we first recovered the lost information of the existing decoy-state method when detecting the weaker version of a PNS attack in the decoy-state MDI-QKD and extracted a normal distribution statistic via statistical hypothesis testing. Based on this information, we proposed a new method to detect the weaker version of a PNS attack. Most importantly, the error probability of detection was precisely calculated by our method, and we also gave the calculation. Finally, according to the judgment result, the corresponding secure key rate was provided. In particular, compared with the existing decoy-state MDI-QKD protocols, the secure key rate with our method has been highly improved if the judgment result is no weak PNS attack. Meanwhile, the associated experimental results also verified the correctness of our method.

Nevertheless, all judgment results in this paper were obtained under the condition that the null hypothesis was no weak version of a PNS attack. In other words, we assume that the gain of signal or decoy state will change significantly after the PNS attack. However, we can do nothing about the stronger PNS attack, which retains the gain of signal and decoy state, such as a partial PNS attack [15], because the premise of the derivation no longer holds, and the Type II error probability of our method in this case will be poor even close to unity. For this reason, compared with the existing decoy-state method [29,30,31] to directly estimate the secure key rate, our method is not ready for practical application now; however, we provide a new direction to improve the secure key rate and efficiency.

## Figures and Tables

**Table 1:** Experimental parameters used in this paper. Data from *Phys. Rev. Lett.* **2014**, *112*, 190503.

$\mu_{2}\left(\nu_{2}\right)$	$\mu_{1}\left(\nu_{1}\right)$	$\mu_{0}\left(\nu_{0}\right)$	$P_{\mu_{2}}\left(P_{\nu_{2}}\right)$	$P_{\mu_{1}}\left(P_{\nu_{1}}\right)$	$P_{\mu_{0}}\left(P_{\nu_{0}}\right)$	$P_{Z}\left(P_{X}\right)$
0.3	0.1	0.01	0.2	0.45	0.35	0.5
$N_{\mathrm{data}}$	$e_{d}$	$e_{0}$	$p_{d}$	$L_{\mathrm{ac}}\left(L_{\mathrm{bc}}\right)$	δ	θ
$1.69\times 10^{11}$	0.01	0.5	$5\times 10^{-5}$	5	0.2	0.8

**Table 2:** The values of $Q_{\mu_{k}\nu_{l}}^{W}\left(\times 10^{-4}\right)$ with intensities $\mu_{k}\in \left\{\mu_{2},\mu_{1},\mu_{0}\right\}$ and $\nu_{l}\in \left\{\nu_{2},\nu_{1},\nu_{0}\right\}$ based on W∈{X,Z}. Reprinted/adapted with permission from Ref. [36], 2014, American Physical Society.

		*Z*			*X*		
	$\mu_{k}$	$\mu_{2}$	$\mu_{1}$	$\mu_{0}$	$\mu_{2}$	$\mu_{1}$	$\mu_{0}$
$\nu_{l}$							
$\nu_{2}$		0.4643	0.1596	0.0215	0.9086	0.4074	0.2449
$\nu_{1}$		0.1596	0.0539	0.0066	0.4074	0.1039	0.0319
$\nu_{0}$		0.0215	0.0066	0.0007	0.2449	0.0319	0.0012

**Table 3:** The values of $n_{\mu_{k}\nu_{l}}^{W}\left(\times 10^{4}\right)$ with intensities $\mu_{k}\in \left\{\mu_{2},\mu_{1},\mu_{0}\right\}$ and $\nu_{l}\in \left\{\nu_{2},\nu_{1},\nu_{0}\right\}$ based on W∈{X,Z}.

		*Z*			*X*		
	$\mu_{k}$	$\mu_{2}$	$\mu_{1}$	$\mu_{0}$	$\mu_{2}$	$\mu_{1}$	$\mu_{0}$
$\nu_{l}$							
$\nu_{2}$		787.5	270.4	38.03	1526	692.9	429.3
$\nu_{1}$		262.0	89.74	11.83	670.9	172.4	52.73
$\nu_{0}$		36.17	11.32	1.521	415.7	53.57	2.366

**Table 4:** The values of $U_{\mu_{k}\nu_{l}}^{W}$ with intensities $\mu_{k}\in \left\{\mu_{2},\mu_{1},\mu_{0}\right\}$ and $\nu_{l}\in \left\{\nu_{2},\nu_{1},\nu_{0}\right\}$ based on W∈{X,Z}.

		*Z*			*X*		
	$\mu_{k}$	$\mu_{2}$	$\mu_{1}$	$\mu_{0}$	$\mu_{2}$	$\mu_{1}$	$\mu_{0}$
$\nu_{l}$							
$\nu_{2}$		1.026	0.6174	3.709	2.415	2.512	10.00
$\nu_{1}$		−7.100	−3.187	4.016	−10.05	−5.452	−3.197
$\nu_{0}$		−0.3709	1.004	5.438	1.209	−0.9135	4.154

## Data Availability

The data presented in this study are available within the article.
